# Research trends among new investigators at ISOQOL: a bibliometric analysis from 2019 to 2023

**DOI:** 10.1186/s41687-025-00878-1

**Published:** 2025-05-14

**Authors:** Jae-Yung Kwon, Manraj N. Kaur, Ellen B. M. Elsman, Ava Mehdipour, Lori Suet Hang Lo, Ahmed M. Y. Osman, Sandrine Herbelet, Carrie-Anne Ng, Lotte van der Weijst, on behalf of the New Investigators Special Interest Group Members

**Affiliations:** 1 School of Nursing, University of Victoria, HSD Building A402A, PO Box 1700 STN CSC, Victoria, BC V8W 2Y2 Canada; 2 Institute on Aging and Lifelong Health, Victoria, Canada; 3 Brigham and Women’s Hospital, Harvard Medical School, Boston, USA; 4 Department of Epidemiology & Data Science, Amsterdam Public Health Research Institute, Amsterdam, Netherlands; 5 School of Nursing, Trinity Western University, Langley, BC Canada; 6 Faculty of Nursing, University of Alberta, Edmonton, Canada; 7 Department of Pharmacy Practice, Kulliyyah of Pharmacy, International Islamic University Malaysia, Kuantan, Malaysia; 8 Department of Head and Skin, Ghent University and Ghent University Hospital, C. Heymanslaan 10, Ghent, 9000 Belgium; 9 Centre for Health Economics Research and Evaluation, Faculty of Health, University of Technology, Sydney, NSW Australia; 10 Quality of Life Department, European Organisation for Research and Treatment of Cancer, Brussels, Belgium

**Keywords:** Quality of life, Bibliometric analysis, Early career investigator, Citation patterns, Research collaboration, Health service research

## Abstract

**Background:**

New investigators (NI), encompassing graduate students, recent doctoral graduates, and early-career faculty, are instrumental in advancing quality of life (QoL) research through innovative methodologies and diverse perspectives. Within the International Society for Quality of Life Research (ISOQOL), the New Investigators Special Interest Group (NI-SIG) fosters collaboration and supports this community. This study utilizes bibliometric analysis to examine the contributions of NI-SIG members, focusing on publication trends, collaboration patterns, and thematic developments in QoL research.

**Methodology:**

Data on publications authored by 56 NI-SIG members between 2019 and 2023 were extracted from Web of Science and Scopus. A two-step screening process, guided by the Wilson and Cleary model of QoL, identified 561 unique documents for analysis. Descriptive metrics included publication trends, citations, journal impact factors, and geographic distribution, while network analysis explored co-authorship patterns. Thematic mapping was conducted using clustering algorithms to identify established and emerging research areas.

**Results:**

Publication output rose steadily from 2019 to 2022, peaking at 163 publications before declining to 135 in 2023, accompanied by a reduction in average citations per document from 4.8 to 1.3. The majority of publications appeared in leading journals such as *Quality of Life Research* (*n* = 128), *Journal of Patient-Reported Outcomes* (*n* = 17), and *BMJ Open* (*n* = 15). Geographic analysis revealed that most contributors were from high-income countries, with the United States, Canada, and Australia accounting for over 50% of publications. Co-authorship network analysis highlighted a robust, interconnected cluster of authors, though opportunities remain to enhance global partnerships, particularly with low- and middle-income countries. Thematic analysis identified well-established areas, including psychometric validation and cancer, alongside emerging topics such as mixed methods in QoL research.

**Conclusion:**

This study highlights robust collaborations among NI-SIG members while identifying opportunities to enhance international collaboration and methodological innovation. Expanding partnerships with underrepresented regions and embracing advanced technologies such as natural language processing could foster inclusivity and drive transformative advancements in QoL measurement and application.

## Introduction

The field of quality of life (QoL) research is dynamic and continually shaped by advancements in theoretical frameworks, changes in cultural and social norms and innovations in technology [1–4]. QoL, as a multidimensional concept, extends beyond traditional health outcomes to encompass physical, mental and social well-being [5]. Its assessment has become a cornerstone of clinical trials, health policy evaluations, and healthcare delivery, reflecting an increasing focus on person-centred care [6]. New investigators (also known as emerging investigators) in QoL research - such as graduate students, recent doctoral graduates, and early-career researchers - play a pivotal role in advancing the QoL field. Their innovative methodologies and novel perspectives have substantially contributed to its growth and diversification [7].

The International Society for Quality of Life Research (ISOQOL) serves as a global platform for advancing QoL research, bringing together over 1,000 members from various sectors such as academia, clinical institutions, regulatory agencies, consultancy, pharmaceutical companies, and biotechnology industries. Within ISOQOL, the New Investigators Special Interest Group (NI-SIG) represents a vibrant and engaged community dedicated to collaboration and shaping the future of QoL research. Recognizing the contributions of new investigators is critical to understanding their impact on QoL research. This study leverages bibliometric analysis to examine the publication trends and collaborative patterns of NI-SIG members, providing insights into emerging areas of interest and fostering engagement. Bibliometric analysis is a well-established method for quantitatively assessing scholarly output [8, 9]. It has been widely used across various scientific and applied disciplines to evaluate research trends, track citation patterns, and map collaboration networks [7, 10, 11]. By systematically analyzing publication data, bibliometric methods provide valuable insights into current trends in the literature and the contribution of individual scholars. In this study, bibliometric analysis is applied to assess the scholarly contributions of NI-SIG members, highlighting emerging research directions and collaborative patterns. These insights aim to create opportunities for knowledge exchange within the broader QoL community, support the professional development of new investigators, and promote global partnerships that drive innovation and progress in QoL research.

## Methods

### Study population

A list of current NI-SIG members was obtained from the ISOQOL, initially comprising 76 members. Since NI-SIG membership is self-selected, we applied the following ISOQOL-defined inclusion criteria to ensure alignment with the New Investigator designation: (a) graduate students, (b) recent doctoral graduates, and/or (c) early-career faculty within five years of earning their final degree. Members were excluded if they exceeded the five-year post-degree limit or if no information was available regarding their degree status (*n* = 15), or if their publication record could not be retrieved (*n* = 5), resulting in a final sample of 56 eligible members.

### Data selection process

All publications in the last five years (i.e., 2019–2023) (co-)authored by the 56 NI-SIG members were extracted from two academic databases: Web of Science (WoS) and Scopus. The five-year window aligns with the ISOQOL definition of a New Investigator to ensure the analysis captures research contributions that reflect the current work of those still in the early-career phase. These databases were selected for their reputable indexing and comprehensive coverage across a wide range of disciplines, including biomedical research and social sciences [12, 13]. The data search was carried out from April 20th to April 24th, 2024. Each NI-SIG member’s first and last names were entered into both databases to retrieve all relevant publications. The search was limited to documents published in English between January 1, 2019, and December 31, 2023.

**Fig. 1:**
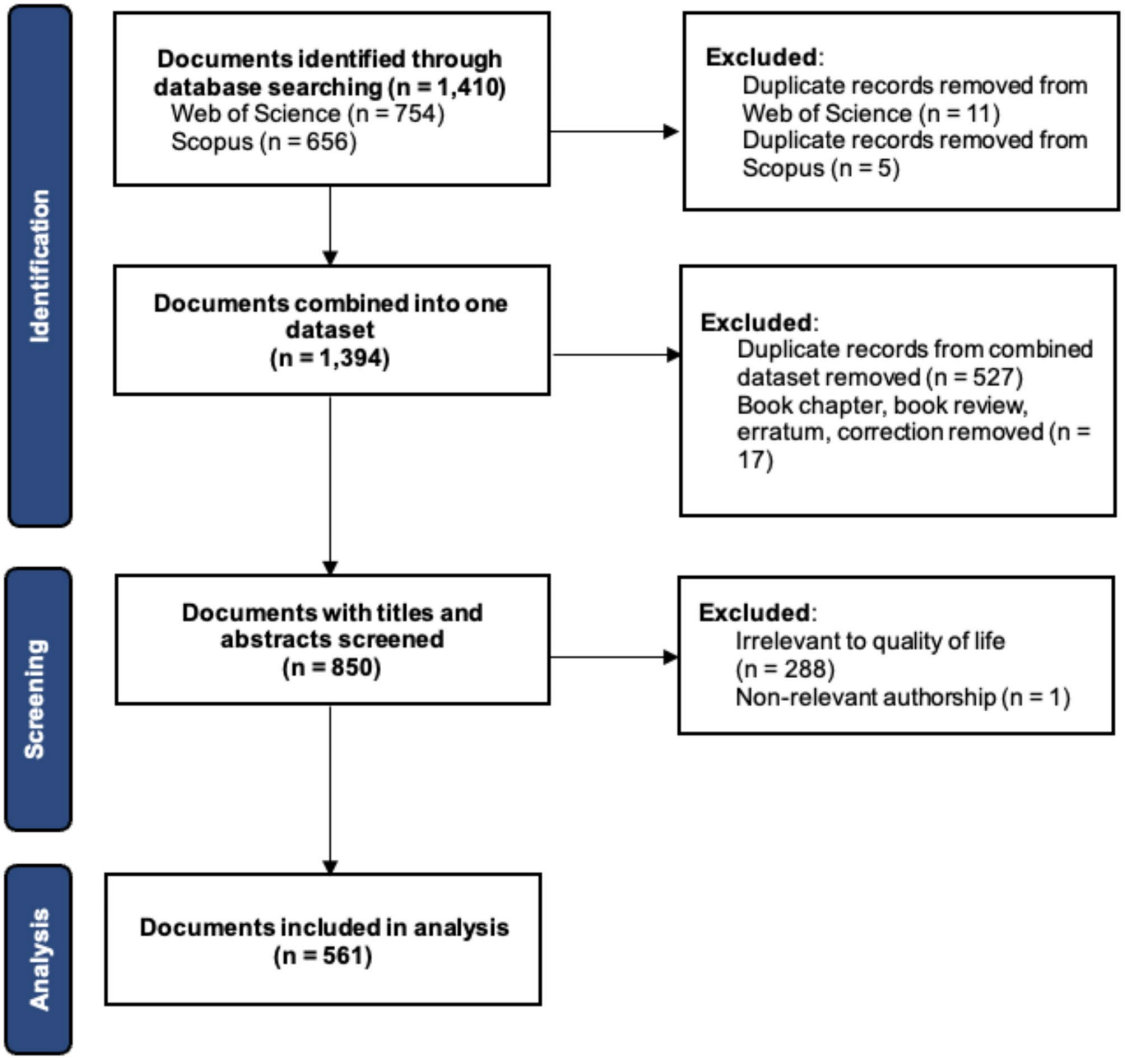
PRISMA Flow diagram of the review process

Initially, a total of 754 documents were retrieved from WoS and 656 from Scopus. After removing duplicate records within each database—11 duplicates from WoS and 5 from Scopus—a combined total of 1,394 documents were obtained (see Fig. 1 of the review process). The datasets were then merged, retaining only common fields such as author names, affiliations, journal titles, document titles, abstracts, document types, and total citation counts. Duplicates within the merged datasets were removed, with preference given to records with higher citation counts, resulting in a refined set of 867 unique documents. We further excluded documents that did not constitute original research, such as book chapters, book reviews, errata, and corrections, which narrowed the dataset to 850 documents. A two-step screening process was then applied to identify studies that specifically addressed QoL as a research topic:

#### Screening for consistency and training

Six team members, who are co-authors of this study (JY, MK, EE, AM, LL, AO, LW), screened the titles and abstracts of a training set containing 3% of the documents. This training set aimed to align the team members’ understanding of the eligibility criteria and ensure consistency in identifying relevant QoL research documents. A high agreement rate of 98% was achieved (calculated as the number of aligned agreements divided by the total). The Wilson and Cleary model served as the guiding framework for defining QoL in this study [14]. This model integrates biological, symptom, functional, and general health perception factors to clarify the relationship between physical health and subjective well-being, offering a comprehensive approach to assessing health status.

#### Independent review and consensus

After reaching consensus on the training set, the remaining 822 documents were independently evaluated by pairs of reviewers, with each pair assessing an equal subset of documents (*n* = 274). Discrepancies between reviewers were resolved through discussion and, when necessary, reviewed by a third team member. This process resulted in a high agreement rate of 91%.

### Analyses

A descriptive analysis was performed to examine publication trends, citation counts, and journal distributions of NI-SIG members. Journal impact factors and subject categories were obtained from Journal Citation Reports, and the results were visualized using Microsoft Excel 2024^®^. To further explore the collaborative relationships among NI-SIG members and their co-authors, a co-author network analysis was conducted. Initially, bipartite edges were first established to link authors with their respective publications. A bipartite edge represents a connection between two distinct sets of nodes, such as authors and the articles they have co-authored [15]. These bipartite edges were subsequently transformed into unimodal edges, which represent direct author-to-author collaborations. This transformation was achieved using matrix multiplication to consolidate the two sets of nodes into a single set representing authors only [16]. In the final network, each node representing an author was weighted according to the logarithmic count of documents. This standard approach normalizes differences in publication volume and reduces skewness caused by highly prolific authors [17]. An adjustment of 0.5 was applied to ensure visual differentiation among authors with fewer publications. The visualization was created in R (version 4.4.0, R Foundation for Statistical Computing, Vienna, Austria) using the *network* package [18].

For the geographic analysis, NI-SIG member institutions were plotted on a world map using the *rnaturalearth* package [19] in R. Geographic coordinates (longitude and latitude) were used to locate institutions, while Gross Domestic Product (GDP) per capital data, sourced from the *World Development Indicator* (WDI) package [20], provided an economic context for each country. To prevent double counting, in cases where two or more NI-SIG members contributed to the same publication, only the member listed first was included.

Thematic mapping was utilized to identify research hotspots and track evolving trends, thereby highlighting areas of concentrated research activity and emerging interests among NI-SIG members. This analysis utilized a hybrid approach integrating author keywords, Keyword Plus, and document titles to provide a comprehensive overview. Author keywords are selected by the original author to represent the content of the publication, whereas Keyword Plus is an automated indexing method that identifies recurring phrases from document titles to uncover additional relevant topics [21]. Since up to 43% of author keywords and Keywords Plus data were unavailable, document titles were prioritized to enhance coverage and improve thematic identification. To enhance clarity and reduce redundancy, synonymous terms were consolidated (e.g., “patient-reported outcome,” “patient-reported outcomes,” and “PRO instruments”). The thematic map was generated using the *biblioshiny* package in R [22], employing a bigram language model option using a Walktrap algorithm. The bigram language model, which identifies sequences of two words, was employed to capture meaningful multi-word expressions that enhance the contextual understanding of topic groupings. To identify clusters of strongly interconnected terms, the Walktrap algorithm, a community detection method based on random walks, was used to construct the thematic map [23]. Parameters were configured to display one label per cluster, with a minimum of 650 words and a cluster frequency threshold of at least 13 occurrences per thousand documents. These values were chosen to maintain a balance between thematic granularity and interpretability. The clustering threshold ensures that identified themes are robust and not statistical anomalies, while the word limit prevents excessive fragmentation of topic themes [24]. These themes are characterized by two properties: density and centrality [25]. Density, represented on the vertical axis, measures the cohesiveness of a theme within the research network, indicating its internal strength and development. Centrality, shown on the horizontal axis, measures a theme’s connectivity to other themes, reflecting its significance and influence in the field. The combination of density and centrality allows for the classification of themes into four quadrants, though these categories are not mutually exclusive, as themes may evolve over time or exhibit characteristics of multiple quadrants, as described by Cobo et al. [24]:

1. Niche themes (top left): High density, low centrality. Specialized themes with strong internal development but limited influence on other themes.
2. Motor themes (top right): High density, high centrality. Well-developed and crucial themes that drive the field forward.
3. Basic themes (right bottom): Low density, high centrality. Broadly connected themes that are foundational but may lack internal development.
4. Emerging or declining themes (left bottom): Low density, low centrality. Themes in the early stages of development or those losing relevance.

## Results

The final dataset was comprised of 561 unique documents, categorized as follows: 281 peer-reviewed articles, 145 meeting abstracts, 108 reviews, 23 commentaries or reports, and 4 proceeding papers (see Fig. 1).

### Publication trends

**Fig. 2:**
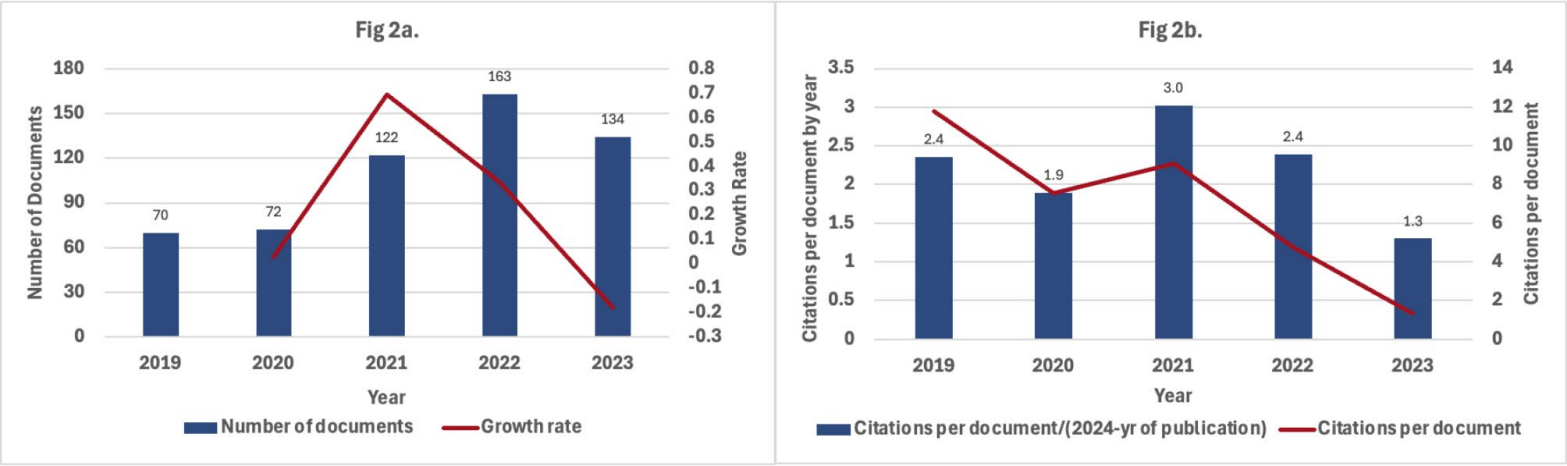
Trends in published documents by new investigators (**a**) and citations per document (**b**) from 2019 to 2023

Figure 2 highlights trends in the number of publications and their citations between 2019 and 2023. As shown in Fig. 2a, the number of publications steadily increased from 70 in 2019 to a peak of 163 in 2022, representing a growth rate of 69% between 2020 and 2021 and a further moderate rise of 34% in 2022. However, this growth momentum declined sharply in 2023, with the total number of publications dropping to 134, resulting in a negative growth rate of 18%. Figure 2b shows the trend in citations per document, adjusted for the number of years since publication (calculated as 2024 minus the publication year). A downward trend is evident, with the decline becoming more pronounced from 2021 to 2023.

### Journal analysis

Included documents were published in 260 journals. Table 1 highlights the top 25 journals with the highest number of published documents by NI-SIG members. Most documents were published in *Quality of Life Research* (*n* = 128, 22.8%), *Journal of Patient-Reported Outcomes* (*n* = 17, 3%), and *BMJ Open* (*n* = 15, 2.7%). In terms of journal impact factor, the *Journal of Clinical Oncology* had the highest five-year impact factor (37.4), with 7 documents published during this period. In addition, *Health and Quality of Life Outcomes* had the highest citations per document (CPD) of 28.5, despite having a smaller number of publications (*n* = 4). The journals spanned variety of disciplines including oncology, rehabilitation, public health, and medical informatics.

**Table 1:** Journals with the highest number of documents on QoL published by new investigators (≥ 0.01%)

Journal name	Peer-reviewed	Meeting abstract	Total documents (%)	CPD	JCR categories	IF (5 year)
Quality of Life Research	30	98	128 (22.8)	1.9	HCS&S; HP&S;P,E&OH	4.4
Journal of Patient-Reported Outcomes	17	0	17 (3.0)	7.1	HCS&S; HP&S	3.1
BMJ Open	15	0	15 (2.7)	8.8	M, G&I	2.7
Acta Ophthalmologica	3	4	7 (1.2)	3.0	Ophthalmology	3.3
Journal of Clinical Oncology	2	5	7 (1.2)	1.1	Oncology	37.4
PLOS One	7	0	7 (1.2)	8.7	Multidisciplinary Sciences	3.3
Journal of Medical Internet Research	6	0	6 (1.1)	3.7	HCS&S; Medical Informatics	6.7
Annals of Surgical Oncology	5	0	5 (0.9)	6.8	Oncology; Surgery	4.0
Blood	0	5	5 (0.9)	0.8	Hematology	19.2
Journal of Cancer Survivorship	5	0	5 (0.9)	12.4	HCS&S; Oncology; Rehab	4.1
Supportive Care in Cancer	5	0	5 (0.9)	8.4	HCS&S; Oncology; Rehab	3.2
Value in Health	4	1	5 (0.9)	9.0	HCS&S; HP&S; Econ	5.6
American Journal of Occupational Therapy	2	2	4 (0.01)	1.8	Rehab	3.2
BMC Health Services Research	4	0	4 (0.01)	7.8	HCS&S	3.1
British Journal of Dermatology	4	0	4 (0.01)	9.0	Dermatology	9.4
Cancers	4	0	4 (0.01)	4.8	Oncology	4.9
Health and Quality of Life Outcomes	4	0	4 (0.01)	28.5	HCS&S; HP&S	3.9
International Journal of Environmental Research and Public Health	4	0	4 (0.01)	8.8	P, E&OH	4.8
Investigative Ophthalmology and Visual Science	1	3	4 (0.01)	1.8	Ophthalmology	4.9
JAMA Dermatology	4	0	4 (0.01)	6.0	Dermatology	10.7
Medical Decision Making	4	0	4 (0.01)	7.9	HCS&S; HP&S; Medical Informatics	3.3
Ophthalmic and Physiological Optics	4	0	4 (0.01)	6.0	Ophthalmology	3.5
Pediatric Blood and Cancer	1	2	4 (0.01)	9.0	Oncology; Hematology; Pediatrics	2.9
Radiotherapy and Oncology	1	2	4 (0.01)	2.0	Oncology; R,NM&MI	5.4
Scientific Reports	4	0	4 (0.01)	6.3	Multidisciplinary Sciences	4.3

Note: CPD = Citations Per Document; JCR = Journal Citation Reports; IF = Impact Factor; HCS&S = Health Care Sciences & Services; HP&S = Health Policy & Services; P, E&OH = Public, Environmental & Occupational Health; M, G&I = Medicine, General & Internal; R, NM&MI = Radiology, Nuclear Medicine & Medical Imaging

### Geographic analysis

**Fig. 3:**
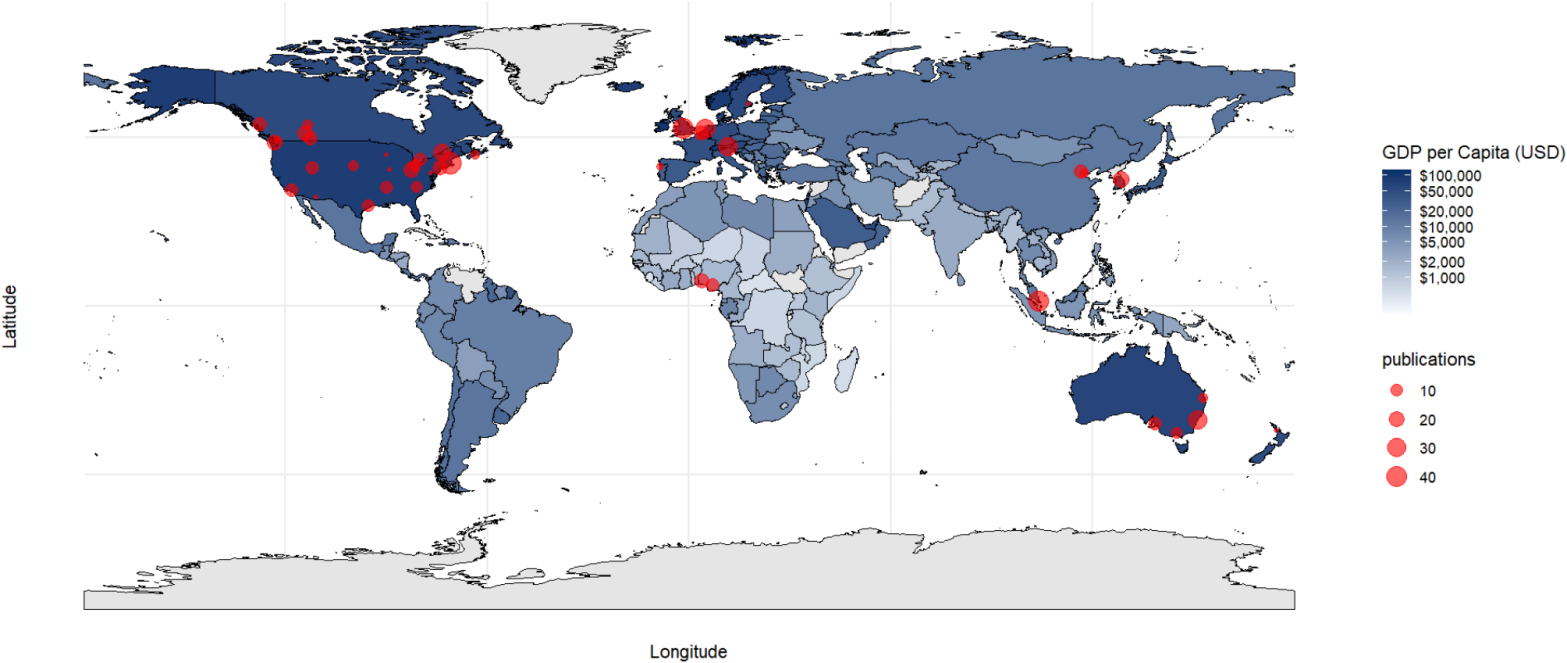
Geographic distribution of new investigators

Figure 3 illustrates the geographic distribution of NI-SIG members, with each red dot representing a unique research institution. The size of each dot corresponds to the institution’s total publication volume, providing a visualization of the global distribution and research productivity. Among the 14 countries represented, the majority of NI-SIG members were located in the United States, with 18 members contributing to 171 publications, accounting for 30.5% of the total output. Canada ranked second, with 16 members producing 106 publications (18.9% of the total). Australia, with 5 members, emerged as another large contributor with 52 publications (9.3%), while Austria, despite having only 1 member, contributed 30 publications (5.3%). Similarly, China and Belgium, with 2 members each, contributed 19 (3.4%) and 16 (2.9%) publications respectively.

### Co-author network analysis

**Fig. 4:**
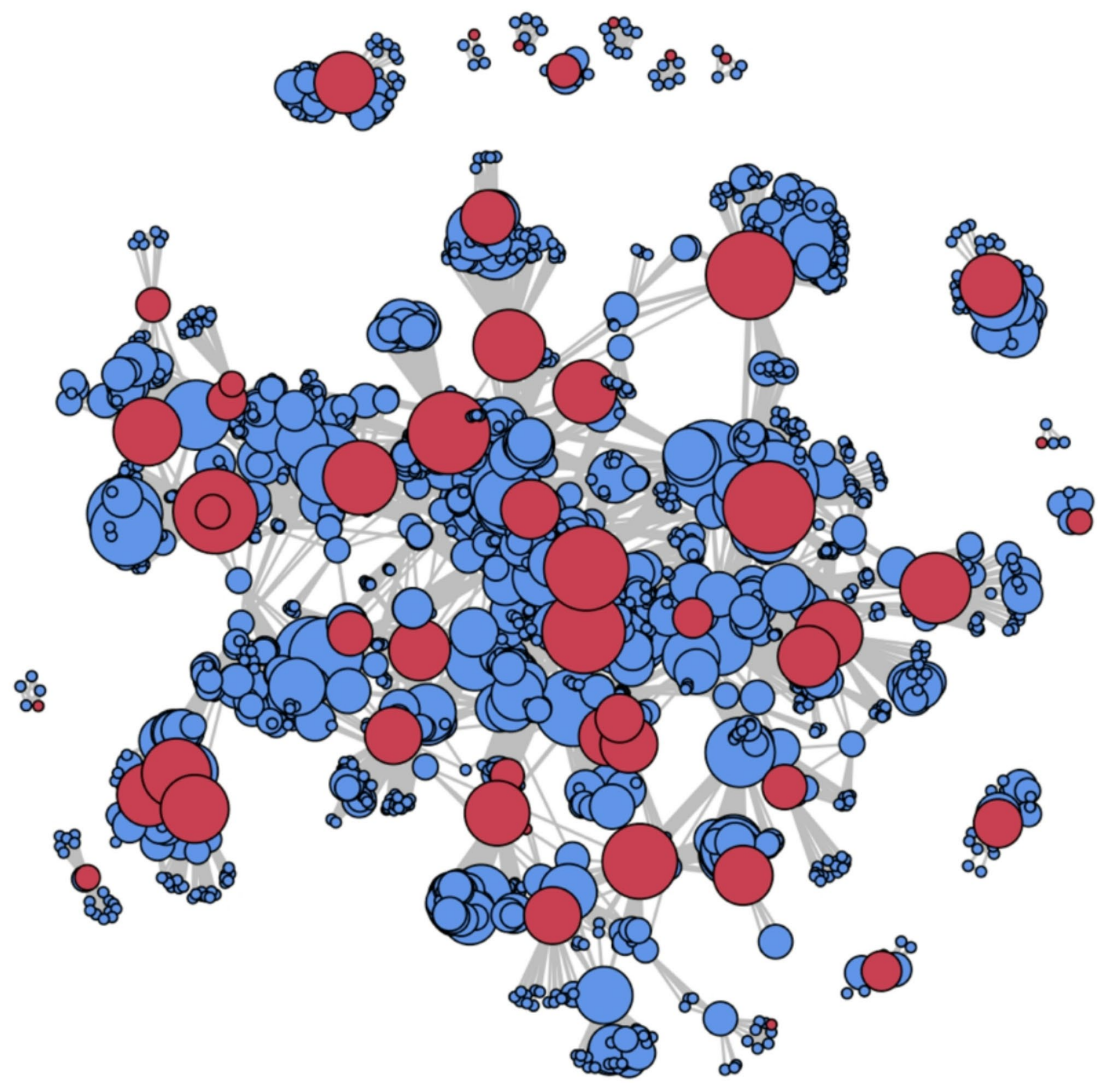
Network analysis of co-authorship with new investigators. Note: Each red node represents an individual new investigator. Blue nodes represent co-authors that are not classified as new investigators

Figure 4 illustrates the co-authorship network, where each node represents an author, and the size of the node represents the number of documents they have published. Links between the nodes indicate co-authorship publications, signifying collaborative relationships between authors. When authors publish together, they form a link in the network, representing mutual collaboration. Overall, there was a large, interconnected cluster involving 42 NI-SIG members and 14 smaller, discrete clusters each involving a single NI-SIG member.

### Thematic map analysis

**Fig. 5:**
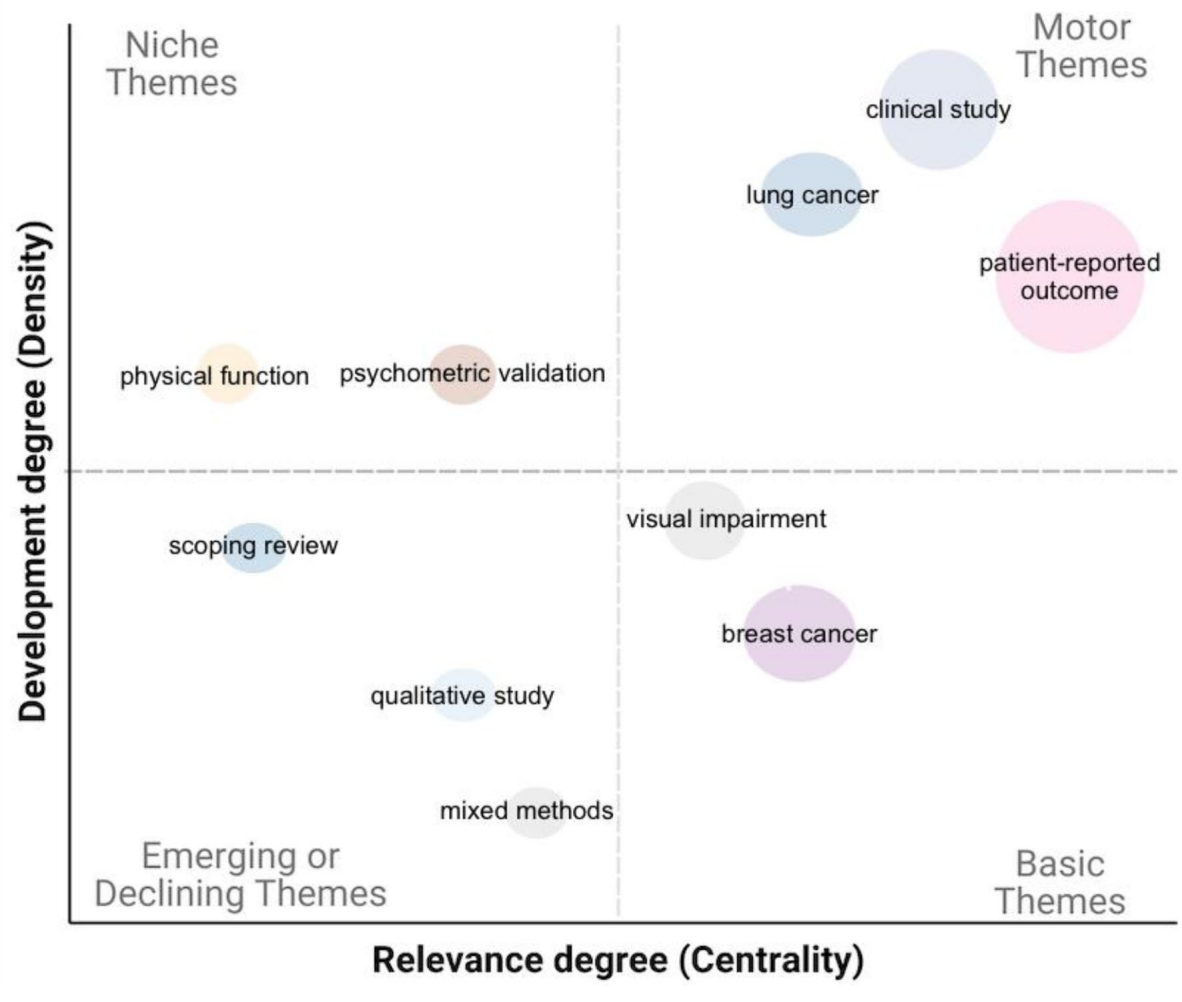
Thematic map using Walktrap for clustering; the size of each node on the map reflects the total number of publications, with centrality indicating how interconnected a theme is within the research network, and density reflecting the internal cohesiveness of the research focus

Figure 5 presents a thematic map based on centrality and density metrics, utilizing Walktrap clustering to identify research themes. The motor theme cluster, which includes “patient-reported outcome”, “clinical study”, and “lung cancer” demonstrates strong research development (318 documents) indicating both high centrality and density. Basic themes such as “breast cancer” and in part “visual impairment” are highly central, underscoring their interconnected role with other themes in the field (51 documents). In contrast, niche themes such as “physical function” and “psychometric validation” are characterized by high density but lower centrality, indicating a specialized theme that is less connected to the broader research landscape. Emerging themes such as “mixed methods”, “qualitative study”, and “scoping review” show higher centrality but moderate density, indicating that they are interconnected with other themes and potentially represent growing areas of interest.

## Discussion

This bibliometric analysis provides significant contributions and evolving trends in research outputs of NI-SIG members between 2019 and 2023 within the field of QoL research, revealing both strengths and opportunities for growth. We observed a strong alignment between NI-SIG research priorities and the focus areas of a few journals. Publications also spanned a wide variety of subject categories, reflecting the multidisciplinary nature of QoL research and its relevance across various fields of healthcare and science. Most NI-SIG members were affiliated with high-income countries and were part of a large cluster of interconnected authors. Established research areas include psychometric validation and cancer, as well as emerging topics.

In terms of publication trends, the analysis identified a notable growth in publications peaking in 2022, with a slight decline in 2023. This fluctuation may partly reflect a backlog of publications resulting from the COVID-19 pandemic [26]. During periods when research activities were disrupted, many researchers shifted their focus toward manuscript preparation, leading to a surge in journal submissions. The peak observed in 2022 likely indicates journals processing this influx of delayed research outputs. The subsequent decline in 2023 may suggest a return to pre-pandemic levels of research productivity. The decline was also evident in the average citations per document for 2023 publications. Although our analysis adjusted for the number of years since publication, newer publications naturally experience a lag in accumulating citations, as it takes time for research to gain visibility and be cited within the academic community. Thus, the lower citation counts for 2023 are likely due to this inherent lag rather than a decrease in research relevance [27]. In addition, shifting research priorities, funding reallocation, and broader global challenges such as the continued impact of the COVID-19 pandemic may have further influenced recent research outputs [26, 28]. For example, the pandemic led to a temporary redirection of funding toward urgent health-related topics, potentially affecting the volume of publications in other fields. It remains to be seen whether the observed decline in 2023 represents a short-term variation or if it will stabilize or reverse as newer publications gain visibility and accumulate citations over time.

The journal distribution data revealed that most publications by NI-SIG members were concentrated in a small number of highly relevant journals, such as *Quality of Life Research* and *Journal of Patient-Reported Outcomes*, both of which are ISOQOL journals. This focus on a few core journals underscores the importance of these outlets in the QoL research community, providing a platform for new investigators to disseminate their work. At the same time, the concentration of publications within a limited number of journals highlights an opportunity to diversify publication avenues. Targeting journals across related domains, such as public health, informatics, and oncology, could enhance the visibility and impact of QoL research, facilitating its dissemination to diverse audiences and encouraging interdisciplinary engagement.

One of the key findings from the co-author network analysis is the presence of a large, interconnected cluster of authors, indicating robust collaborative efforts among some NI-SIG members. This may be attributed, in part, to publishing with mentors or within mentorship teams. Similar patterns of collaboration have been observed in related fields, such as research by new investigators in chronic disease management [29]. This suggests that fostering strong networks may be a common strategy to accelerate knowledge exchange and enhance research impact across various health domains. Future studies could explore whether these collaborative trends extend to other areas, potentially identifying opportunities for cross-disciplinary synergies and shared learnings. However, the analysis also identified geographic and institutional disparities in collaboration patterns, as NI-SIG members were predominantly concentrated in high-income countries. Strengthening international partnerships, particularly with researchers from low- and middle-income countries (LMIC), provides a vital opportunity to integrate diverse perspectives and address global QoL challenges [30]. Evidence indicates that international research collaborations can enhance research quality and impact national collaborations [31–33]. For example, a study analyzing 148,977 UK-based journal articles found that international co-authorship increased the likelihood of higher quality scores in 27 out of 34 research areas [31]. To foster successful global health research partnerships, four key factors have been identified: (1) mutual respect and benefit, (2) trust, (3) good communication, and (4) clear partner roles and expectations [34]. Funding agencies and academic institutions can support these collaborations by prioritizing grant opportunities that encourage international partnerships and develop standards and mechanisms to enhance integration with research consortia and networks [35, 36]. These initiatives help connect researchers globally, providing resources and support to overcome logistical challenges. Capacity-building initiatives like joint training programs, hosting workshops, and exchange visits can empower LMIC researchers to engage more actively in QoL research [37]. By expanding these international partnerships, the research community can enrich the diversity of perspectives, thereby enhancing the global relevance and applicability of findings. In addition, NI-SIG members with fewer collaborations could benefit from connecting with co-authors who are well-connected within academic networks, such as those represented by larger-sized blue nodes in the network analysis.

A noteworthy aspect for further discussion involves NI-SIG members who are not part of the large, interconnected cluster. While further analysis revealed that these members were not predominantly from lower-income countries or engaged in niche research interests, it remains important to explore whether they faced other barriers to integration within the network, such as access to resources, institutional support, or collaboration opportunities. Understanding these dynamics could provide deeper insights into the challenges and opportunities within academic collaboration and help inform strategies to better support new investigators in building robust and inclusive academic networks.

The thematic analysis identified well-established research areas, such as patient-reported outcomes and specific cancers, which continue to be central to the QoL research landscape. However, the absence of certain cancer types, such as metastatic cancer, suggests potential research gaps or areas that may be underexplored by the NI-SIG members. Niche themes, such as psychometric validation, play a critical role in advancing measurement tools and improving the accuracy of QoL assessments in clinical studies. These themes highlight the current ongoing efforts to refine methodologies and ensure the validity and reliability of QoL measures across various contexts. Emerging themes, including mixed methods and qualitative studies, indicate a growing interest in integrating diverse methodological approaches to better understand and assess QoL. These themes reflect an increasing emphasis on capturing the complexities of patient experiences and contextual factors in research. The application of advanced technologies, such as natural language processing, may have the potential to address challenges in analyzing large-scale unstructured data and developing more personalized approaches to health and QoL [38].

Overall, this study highlights the contributions of NI-SIG members to the advancement of QoL research, showcasing their efforts in fostering collaboration and addressing key research areas. At the same time, it underscores the ongoing need for greater international collaboration and a more proactive exploration of emerging research topics to further expand the impact and reach of QoL research. Strengthening global partnerships, particularly with underrepresented regions, could provide valuable opportunities to integrate diverse perspectives and foster innovation within the QoL research community. Initiatives such as capacity-building programs, equitable research funding, and collaborative networks could help bridge gaps and enable researchers from diverse backgrounds to contribute meaningfully. Evidence from prior studies suggests that international collaborations enhance research impact and innovation by combining unique regional expertise and addressing global health challenges collectively [39, 40]. By prioritizing inclusive collaboration frameworks, the QoL research community can ensure broader representation and more comprehensive approaches to addressing complex global issues. Future research should focus on integrating diverse methodological approaches to address the evolving challenges of QoL measurement and application. This includes embracing innovative technologies, such as machine learning and natural language processing, to enhance the precision and personalization of QoL assessments.

### Strengths and limitations

To our knowledge, this is the first study to analyze NI-SIG members within ISOQOL using bibliometric methods to assess their contributions to the literature. By leveraging both WoS and Scopus, we provided a more comprehensive analysis of publication trends, collaboration patterns, and thematic developments. These insights not only enhance our understanding of NI’s impact in QoL research but also offer a more nuanced approach to measuring scholarly output in addition to conventional metrics such as publication counts and citation numbers. Despite these strengths, several limitations should be considered. First, the analysis was confined to publications indexed in WoS and Scopus, which may not encompass all relevant research outputs, especially those published in non-indexed or regional journals. As a result, contributions from emerging regions or niche areas of study may have been underrepresented. To provide a broader perspective, meeting abstracts were included as a complementary data source. These abstracts provide valuable insights into emerging research trends and preliminary findings that may not yet have progressed to full publication. Highlighting preliminary results capture the evolving interests of the scientific community, particularly among new investigators, and complement peer-reviewed articles in capturing the trajectory of research developments. Although efforts were made to minimize duplication, it is possible that some meeting abstracts, which later developed into full peer-reviewed articles with modified titles, were not entirely excluded. Second, given that NI-SIG membership is voluntary, members may systematically differ from non-members, introducing the potential for self-selection bias. While demographic characteristics could offer valuable insights into the representativeness of the NI-SIG membership, such data are typically not available in published records. Moreover, our study utilized a blanket consent approach, which did not collect this information. To enhance the generalizability of our findings, future research should explore ways to integrate sociodemographic data for a more nuanced understanding of early-career researcher representation. Third, the use of author affiliations to determine country data may have led to incomplete or inaccurate geographic information, despite extensive manual verification efforts. In addition, the geographic distribution of publications is closely tied to the institutional affiliations of NI-SIG members, leading to an overrepresentation of new investigators from developed countries. This reflects the broader composition of the NI-SIG membership, where most researchers are affiliated with institutions in high-income countries, limiting the global network represented in this study. Consequently, the findings and recommendations may not fully capture the contributions of NIs from LMICs. Future efforts should focus on fostering international collaborations and expanding participation from LMICs to strengthen the diversity and inclusivity of the NI-SIG network. Enhancing representation from these regions would provide a more comprehensive understanding of global QoL research contributions and support a more equitable exchange of knowledge within the field.

## Conclusion

While new investigators are making significant contributions to the field of QoL research, there remains ample opportunity to enhance their impact through increased international collaborations and engagement with emerging research areas. By expanding their networks and exploring innovative methodologies, the NI-SIG members can help drive the future direction of QoL research, contributing to more comprehensive and inclusive approaches to understanding QoL across diverse populations. The findings from this study provide a valuable foundation for future efforts to support and amplify the work of new investigators, ensuring that their contributions continue to shape the evolving landscape of QoL research.

## Data Availability

The datasets used and/or analysed during the current study are available from the corresponding author on reasonable request.
